# Late preterm and very preterm infants differ in the acquisition time and quantity of reaches with grasping at reaching onset: an exploratory study

**DOI:** 10.3389/fpsyg.2023.1278774

**Published:** 2023-10-27

**Authors:** Andressa Lagoa Nascimento França, Priscila Maier Teruia, Amanda de Oliveira Arguelho, Eloisa Tudella, Daniele Almeida Soares-Marangoni

**Affiliations:** ^1^Graduate Program in Health and Development, Faculty of Medicine, Federal University of Mato Grosso do Sul, Campo Grande, Brazil; ^2^Institute of Health, Federal University of Mato Grosso do Sul, Campo Grande, Brazil; ^3^Graduate Program in Movement Sciences, Institute of Health, Federal University of Mato Grosso do Sul, Campo Grande, Brazil; ^4^Graduate Program of Physical Therapy, Department of Physical Therapy, Federal University of Sao Carlos, São Carlos, Brazil

**Keywords:** premature, child development, motor skills, risk factors, exploratory behavior

## Abstract

**Introduction:**

The onset of manual reaching allows the expansion of the infant’s interaction with the environment. When born preterm, infants become vulnerable to problems in the development of reaching. However, it is still unknown whether there are differences in reaching according to the degree of prematurity.

**Objective:**

This study aimed to explore the differences in reaching acquisition and behavior between late preterm and very preterm infants, as well as whether age and clinical variables influence the results.

**Method:**

This is an exploratory, comparative, observational study. In total, 24 infants were included soon after reaching onset; 12 infants were born late preterm (35.55 ± 0.67 gestational weeks) and 12 very preterm (30.60 ± 0.05 gestational weeks). Infants were placed in a baby seat, and a toy was placed at a reachable distance for 2 min. Reaching behavior was the primary variable; birth weight and length of hospital stay were secondary variables.

**Results:**

The age of reaching onset was higher in the very preterm group. The proportion of reaches with grasping was higher in the late preterm group. These differences were affected by the lower birth weight and longer length of hospital stay in the very preterm group. The proportions of proximal and distal adjustments did not differ between groups.

**Conclusion:**

Very preterm infants presented disadvantages in the acquisition time and the number of reaches with grasping, but not in the proportions of proximal and distal adjustments of reaching, relative to late preterm infants. Group differences were influenced by clinical variables.

## 1. Introduction

Preterm birth (<37 gestational weeks) enforces a challenging adaptation of the newborns to extrauterine life (World Health Organization, 2023). If hospitalization is required, preterm newborns experience several painful and invasive stimuli during procedures necessary for survival but that may be noxious for their immature nervous system (Giachetta et al., 2010). Additionally, the lowest the gestational age and the birth weight, the greatest the chances of mortality and morbidities (Munhoz et al., 2022). Hence, the physical immaturity associated with the exposition to an adverse extrauterine environment puts preterm newborns at an increased risk of developmental problems (Munhoz et al., 2022; Rees et al., 2022). These problems can extend beyond the neonatal period (World Health Organization, 2023).

One of the motor skills commonly affected by preterm birth is manual reaching. The onset of reaching typically occurs at 3–5 months of age and requires the ability to locate the object in space and move one or both hands to finally touch it (Thelen et al., 1993; Cunha et al., 2013). It is considered a fundamental milestone of human development as it expands the infant’s ability to explore the environment in an active and independent way (Lobo and Galloway, 2013). In preterm infants, due to their biological limitations (e.g., poor regulation of muscle strength and learning difficulties), the development of their manual behaviors can be characterized by delays and dysfunctions (Guimarães et al., 2013).

Compared with typically developing full-term infants, very preterm infants (28 to 31/6-week gestation) with low birth weight delay the acquisition of reaching (Fallang et al., 2005; Heathcock et al., 2008) and use less adapted manual strategies to reach for toys at 4 months of age (Heathcock et al., 2008; Grönqvist et al., 2011). In late preterm infants (34 to 36/6-week gestation), compared with full-term infants, no substantial differences have been found regarding reaching and grasping performance from 5 to 7 months of age (Toledo and Tudella, 2008; Toledo et al., 2011). However, late preterm infants delay the period of reaching onset and are less advanced in the selection of proximal (uni- or bimanual reaching) and distal (e.g., hand opening) adjustments of the upper limbs than full-term infants at this early reaching phase (Soares et al., 2014). Early signs of difficulties in reaching performance can predict neuromotor problems that become evident years later (Fallang et al., 2005; Kaul et al., 2019).

Despite the clinical importance of assessing reaching behavior as a potential strategy to monitor developmental problems in preterm infants, no studies that have investigated whether delays and early changes in reaching behavior are different according to degrees of prematurity were found. Furthermore, it is not known whether these problems are influenced by other clinical features. The objective of this study was to explore differences in early reaching behavior between two groups of infants with different degrees of prematurity. The following question guided this study: (a) Do very preterm infants (higher degree of prematurity) differ from late preterm infants (lower degree of prematurity) in the age of acquisition of reaching and in the reaching behavior at this period? (b) Do age and clinical variables, such as birth weight and length of hospital stay, affect the results? The results can expand the knowledge of the impact of preterm birth on early manual skills and guide further research.

## 2. Materials and methods

### 2.1. Study design

This is an exploratory observational study with a comparative analytical cross-section.

### 2.2. Participants

**Figure 1:**
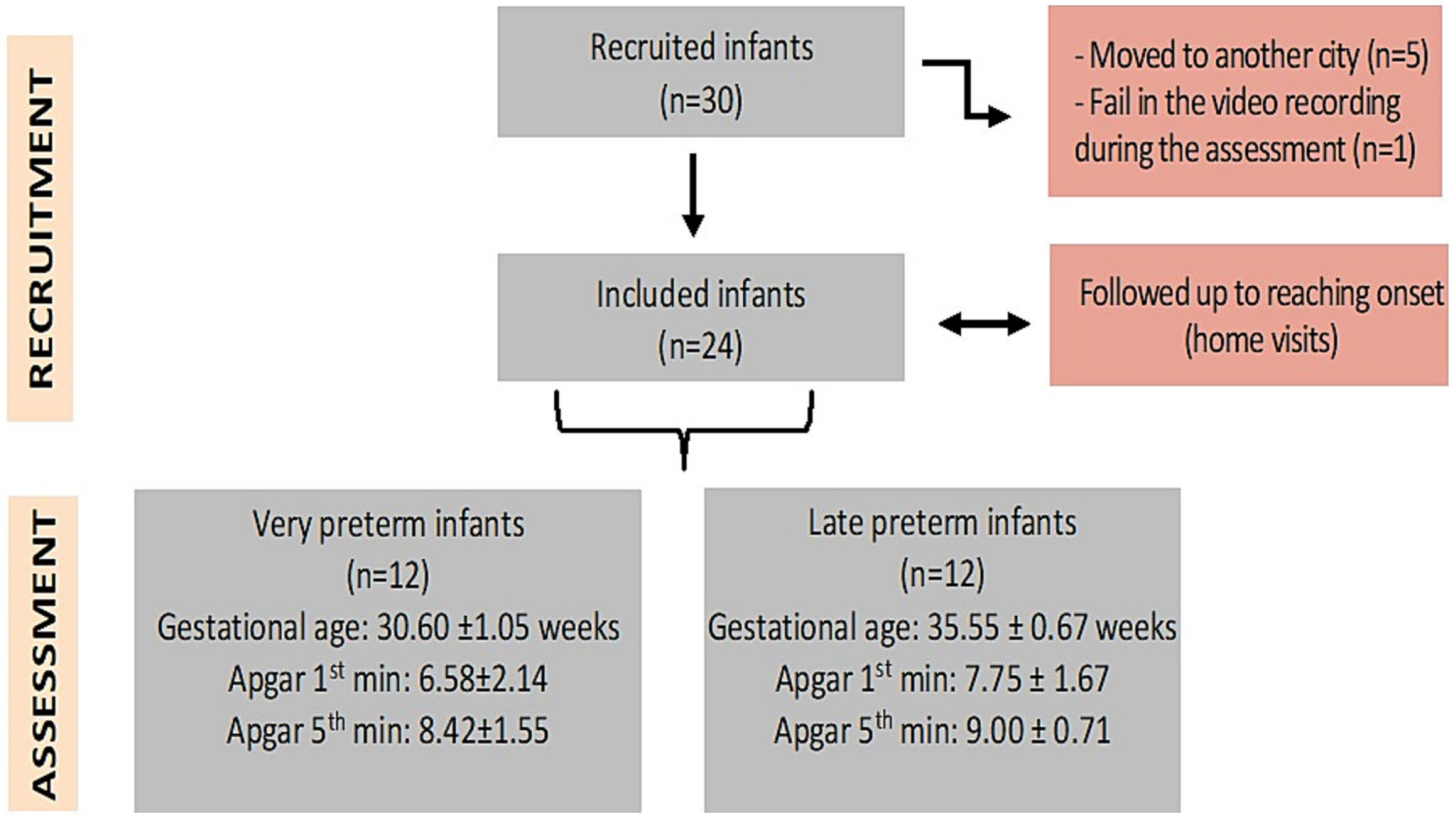
Study design and sample composition. Measures of central tendency and dispersion represent mean and standard deviation.

Participants were 30 preterm infants, recruited from the Neonatal Intermediate Care Unit (NICU) of the University Hospital of the Federal University of Mato Grosso do Sul, Brazil. The infants were assessed between 2016 and 2018. The final sample consisted of 24 infants (12 late preterm and 12 very preterm infants). Infants were assessed immediately after the emergence of reaching (Figure 1).

For inclusion in the study, infants had to be born preterm, with a gestational age of 34 to 36/6 weeks (late preterm) and 28 to 31/6 weeks (very preterm), regardless of birth weight or length of hospital stay in neonatal units. All infants should be under maternal home care. Diagnosis of anoxia, signs of neurological complications, hyperbilirubinemia, congenital malformations, genetic syndromes, progressive neuromuscular conditions, and orthopedic, sensory (auditory and visual), or cardiorespiratory problems were exclusion criteria.

The study was approved by the Human Research Ethics Committee of the Federal University of Mato Grosso do Sul (protocol number 2355473/2017). All parents signed the informed consent form authorizing their infants’ participation.

### 2.3. Procedures

**Figure 2:**
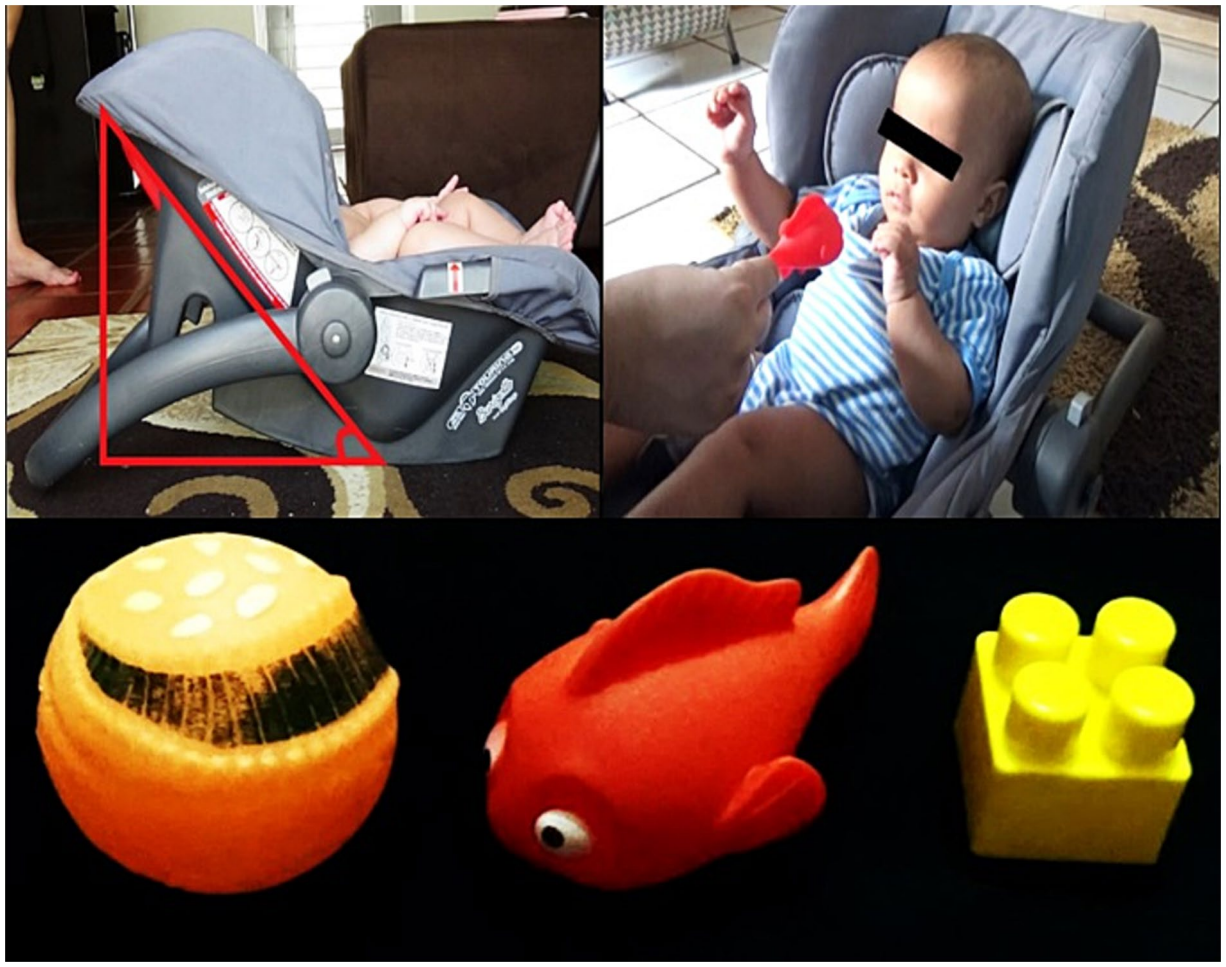
Reaching assessment (upper images): baby seat with 45° inclination in relation to the horizontal axis; an attractive object is offered in the midline of the infant’s body, at the height of the xiphoid process, and at a distance from the length of the infant’s upper limb to the palmar region. Toys used during the reaching assessments (lower image).

To follow up on the emergence of reaching, home visits were carried out by the researcher twice a week from the 12th week of the chronological age of the infants (Soares et al., 2013; Kaul et al., 2019). During the visits, the infant was placed in a baby seat with a 45° inclination in relation to the horizontal axis. Toys were offered in the midline of the infant’s body at his/her xiphoid process height and at a reachable distance (Figure 2). The emergence of the skill was considered when the infant was able to perform three to five reaches within approximately 1 min during the visit (Soares et al., 2013; Nascimento et al., 2019). Reaching assessment was scheduled within 5 days.

Reaching assessment was performed on a single day. Infants should be in an active alert state. The infant was placed in the baby seat, and a toy was presented for 2 min (Rocha et al., 2006; Figure 2). During this period, the toy was carefully taken away and another toy was offered to the infant after each hand contact with the toy (i.e., a reach). The interval between toy presentations was approximately 5 s (Rocha et al., 2006; Nascimento et al., 2019). If the infant did not touch the object, it was taken away in the same time interval to prevent habituation. The toys were the same used in the home visits. They ranged from 4 to 6 cm^2^ in size and varied in shape and texture (rigid plastic cube, soft rubber fish, rubber ball with medium malleability); the goal was to offer varied opportunities for infants to reach and grasp, thus mimicking the actual environment. The toys were presented always in the same sequence for all infants. No verbal or physical encouragement was provided for the infant during the assessments. If the infant was fussy or crying, the assessment was interrupted, the infant calmed down and the assessment restarted.

### 2.4. Description of variables

Clinical variables considered were birth weight (body mass at birth, in grams) and length of hospital stay. These data were collected from the hospital discharge reports provided by the parents. The length of hospital stay was considered the total number of days the infant remained hospitalized in the NICU. The age of emergence of reaching (age at reaching onset) was considered as the one in which the infant was able to perform three to five reaches in 1 min.

The reaching outcomes considered were the total number of reaches, proximal adjustments, distal adjustments, and grasping outcome.

The total number of reaches was considered as the number of valid reaches performed by the infant. A reach was considered valid when the infant performed the movement with one or both upper limbs toward the object until touching it, with or without grasping (Savelsbergh and Van Der Kamp, 1994; Carvalho et al., 2008; Toledo et al., 2011; Soares et al., 2013).

Proximal adjustments were considered as the initiative to direct one or both upper limbs to the presented toys and were categorized as follows: (a) unimanual reaching, when the infant moved only one of the upper limbs toward the object until touching it (Rocha et al., 2006; Barrett and Needham, 2008; Heathcock et al., 2008; Nascimento et al., 2019) or (b) bimanual reaching, when the infant simultaneously moved the upper limbs toward the object; hands should move simultaneously up to at least half of the range of motion (50% of the trajectory) and touching the object could be performed with one or both hands (Corbetta and Thelen, 1996; Rocha et al., 2006; Cunha et al., 2013; Soares et al., 2013).

Distal adjustments were considered as the hand configuration at the object touch, as follows: (a) open hand, when the fingers were fully extended or slightly flexed; (b) closed hand, when all the fingers were completely flexed or, in a few cases, when all the fingers were completely flexed and only one finger was extended; or (c) semi-open hand, when the fingers were in an intermediate position between open and closed (Soares et al., 2013).

The outcome of reaching in terms of grasping was also considered: (a) reaching with grasping, when the infant was able to hold the object or part of it using the hand or fingers of one or both hands after a valid reaching; and (b) reaching without grasping, when the infant reached the object without grasping it (Wimmers et al., 1998; Soares et al., 2013).

### 2.5. Statistical analysis

Statistical analysis was performed using SPSS 23.0. The Shapiro–Wilk test was used to evaluate data distribution. Clinical variables and age were described using mean and standard deviation; median, minimum, and maximum were used to describe reaching outcomes. The total number of reaches were analyzed considering the frequency of their occurrence, and the proximal and distal adjustments and grasping were analyzed considering their proportions in relation to the total number of reaches.

For comparisons between groups (late preterm × very preterm), independent *t*-tests were applied for clinical variables and age. The Mann–Whitney U-test was applied to reach outcomes. Non-parametric ANCOVA (Quade’s test) was used to examine the effect of clinical variables and age as intervening variables on reaching outcomes, considering birth weight, length of hospitalization stay, and age at reaching onset as covariable. The adjusted R^2^ was used to determine the effect size; the stronger its value, the stronger the strength of the association between the dependent variable and the intervening variable (Ialongo, 2016). An α significance level of 5% was adopted for all analyses.

## 3. Results

With respect to clinical variables, birth weight was higher in the late preterm group (2.510 ± 0.34 grams) than in the very preterm group (1.405 ± 0.23 grams; *t* = 8.91; *p* < 0.01). The very preterm group had a longer hospital stay at the NICU (34.67 ± 13.80 days) than the late preterm group (0.0 ± 0.0 days; *t* = −8.33; *p* < 0.01); late preterm infants were not admitted to the NICU.

The age at reaching onset was higher in the very preterm group (5.13 ± 0.59 months chronological age; 4.04 ± 0.61 months corrected age) than in the late preterm group (5.64 ± 0.54 months chronological age; 3.34 ± 0.40 months corrected age) for both chronological (*t* = 2.20; *p* = 0.04) and corrected (*t* = 3.30; *p* = 0.003) ages.

The total number of reaches was similar between the groups (U = 55.50; *p* = 0.35). Bimanual reaches (Med = 71.43%; min–max = 20.00–100.0%) and reaches with semi-open hand (Med = 91.67%; min–max = 33.33–100.0%) were the most adopted proximal and distal adjustments, retrospectively, but there were no differences between the groups for those variables (U’s < 70.50; *p*’s > 0.24; Table 1).

**Table 1:** Reaching age (mean ± standard deviation) and outcomes (median, min-max) in each group.

Reaching	Late preterm	Very preterm
	*n* = 12	*n* = 12
Age at acquisition (days)^*^	153.93 ± 16.86	169.17 ± 15.65
Total number of reaches	9 (4–15)	11.50 (7–15)
Unimanual reaches (%)	26.78 (0.0–77.78)	35.12 (7.69–80.0)
Bimanual reaches (%)	73.21 (22.22–100.0)	64.88 (20.00–92.31)
Open hand (%)	00.0 (0.0–66.67)	00.0 (0.0–27.27)
Semi-open hand (%)	91.61 (33.33–100.0)	91.67 (57.14–100.0)
Closed hand (%)	00.0 (0.0–50.00)	00.0 (0.0–21.43)
With grasping (%)^*^	67.53 (0.0–100.0)	3.85 (0.00–33.33)
Without grasping (%)^*^	32.47 (0.0–100.0)	96.15 (66.67–100.00)

* Differences between groups (*p* < 0.05).

**Figure 3:**
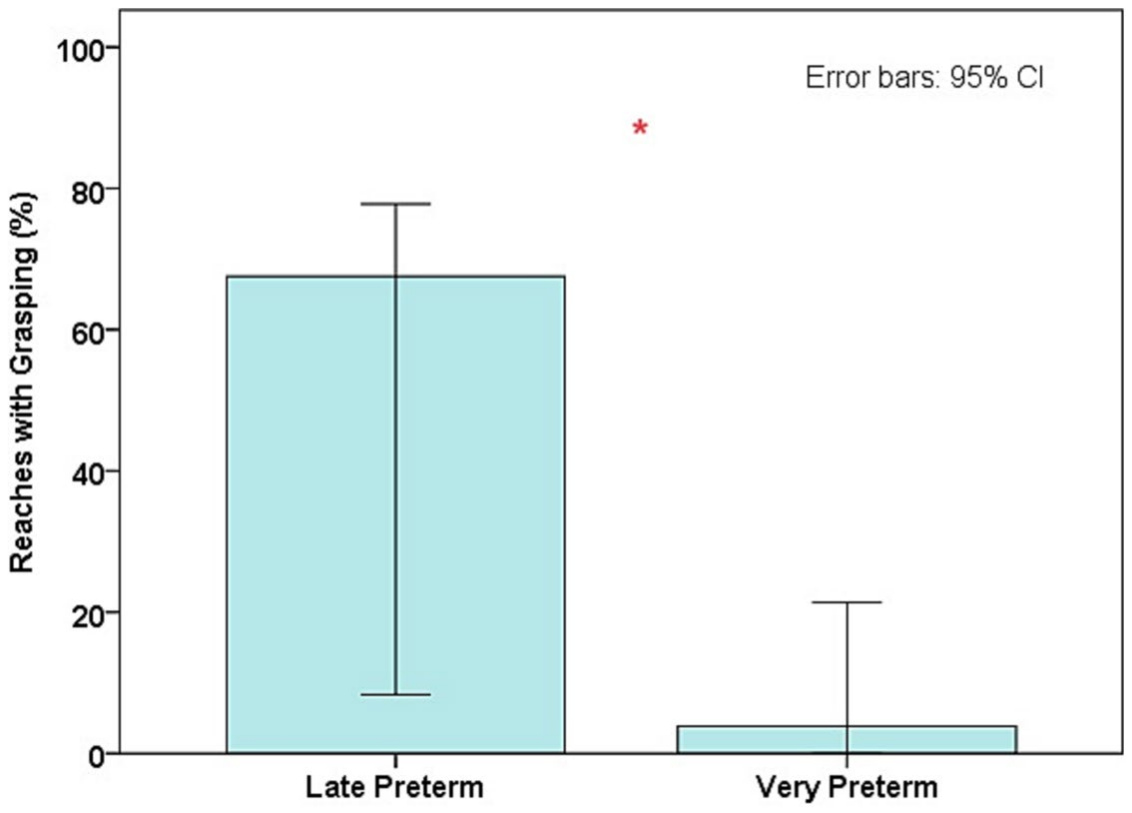
Median values of the proportions of reaches with grasping in both groups; ^*^Differences between groups (*p* < 0.05).

There was a difference between the groups for grasping. The proportion of reaches with grasping was higher in the late preterm group than in the very preterm group (median difference = 63.68%; U = 20.00; *p* = 0.004; Figure 3; Table 1). This result was maintained in the covariance analysis, after adjusting the values for age at reaching onset (F_1,24_ = 10.53; *p* = 0.004; adjusted R^2^ = 0.293), which indicated this variable did not affect the result. The group difference was not maintained when birth weight (F_1,24_ = 1.100; *p* = 0.306; adjusted R^2^ = 0.004) and length of hospital stay were analyzed as covariates (F_1,24_ = 0.30; *p* = 0.590; adjusted R^2^ = 0.03), which indicated that despite the small association, these variables affected the variance in the proportions of grasping.

The other reaching outcomes remained similar between the groups in the covariance analysis after adjusting the values for age at reaching onset (F_1,24_’s > 0.01; *p*’s < 0.92; adjusted R^2^ = 0.031–0.045), birth weight (F_1,24_’s > 0.07; *p*’s < 0.93; adjusted R^2^’s = 0.006–0.045), and length of hospital stay (F_1,24_’s > 0.01; *p*’s < 0.99; adjusted R^2^’s = 0.032–0.045) and indicated small associations between these independent variables and the dependent reaching outcomes.

## 4. Discussion

To the best of our knowledge, this is the first study to address differences in reaching according to the degree of prematurity. In general, we found that very preterm infants had disadvantages in the time of reaching onset and in the number of reaches with grasping compared with late preterm infants.

The acquisition of reaching was 3 to 4 weeks earlier in late preterm infants than in very preterm infants, considering both chronological and corrected ages. This indicates that very preterm infants need more time to learn and execute their first reaching movements. This also suggests that the longer exposure time in the extrauterine environment in the very preterm infants, due to their earlier birth, did not favor spontaneous practice that could improve the skill. Previously, it has been demonstrated that late preterm infants delayed the acquisition of reaching by up to 1 month compared with full-term infants. It was suggested that the additional extrauterine period did not overcome possible limitations associated with brain immaturity at birth and that could influence the process of learning to reach (Soares et al., 2014). The present study extends these findings by demonstrating that such limitations in the reaching emergence process also occur across degrees of prematurity, particularly between very preterm infants and late preterm infants.

It has been also demonstrated previously that late preterm infants had less variability of proximal and distal adjustments available to reach for the object compared with full-term infants in the emergence of reaching (Soares et al., 2014). It was suggested that late preterm infants were less advanced in the process of selecting the most efficient adjustments for reaching. The present study shows that these differences did not occur among the preterm infants studied. Once reaching was acquired, the mean difference of 5 weeks of prematurity between the groups was not enough to determine advantages in spontaneous practice that could favor the number of reaches and adjustments performed by the less premature infants.

The most adopted reaching adjustments in both groups in this study were bimanual and semi-open hand reaches. Bimanual reaching can be a strategy adopted by preterm infants as an effort to reach, given the relatively poor motor control of their muscles (Soares et al., 2013). Reaching with a semi-open hand is a functional strategy to try to grasp the object, but it can be adopted even by less skilled infants (Carvalho et al., 2008). Therefore, both groups may have presented limitations in motor control to perform reaching considering they were born preterm. It is also possible that the sample size was insufficient to evidence differences between the groups. Interestingly, a difference appeared when performing a more advanced skill, as reaches with grasping were performed less by very preterm infants than by late preterm infants.

Object grasping is one of the main outcomes of reaching. The infant moves the hand toward the object usually with the intention of apprehending it. However, reaching that results in grasping is a more complex skill than reaching in itself (Hadders-Algra, 2013), and very preterm infants probably had more difficulty than late preterm infants in dealing with this motor complexity. To grasp an object, in addition to the synergistic activation of the proximal muscles of the upper limb to guide and sustain the limb in the air against the action of the force of gravity (Savelsbergh and Van Der Kamp, 1994; Hadders-Algra, 2013), the distal muscles must contract harmonically to adjust the palmar configuration to grasp the object (Barrett and Needham, 2008; Toledo and Tudella, 2008; Takei and Seki, 2010). This is coordinated based on the infant’s perception of the object, such as positioning and size, by gathering sensory information that guides motor planning and indicates which more efficient movement patterns he/she should select to successfully perform grasping (Gibson, 1986; Lee et al., 2006). This self-organization in grasping becomes even more complex in a period of little experience in manual activities, such as in the first days after the emergence of reaching – when the infants were assessed in the present study. At this stage, grasping is still very immature. As preterm infants may have difficulties in modulating reaching movements and in motor learning processes in the first months of life (Gekoski et al., 1984; Heathcock et al., 2008; Soares et al., 2014), we suggest that the late preterm infants were more advanced than the very preterm infants in the processes of planning and selecting patterns of muscle activation and movements that were more effective for grasping.

Our results also showed that the between-group difference in reaching with grasping disappeared when the influences of birth weight and length of hospital stay were controlled. Hence, despite the small influence of these clinical variables on object grasping, it was enough to affect its outcome. Possibly, such influence might be higher in larger samples. In any case, due to lower weight and greater physiological immaturity at birth, as well as associated morbidities, the very preterm infants remained hospitalized for approximately 20 to 48 days. In contrast, the late preterm infants did not require extra hospitalization, just the usual period of 48 to 72 h after delivery, going home and probably having more chances of exposure to adequate stimuli in a more welcoming environment than the NICU.

Studies have reported that low birth weight (Munhoz et al., 2022) and prolonged stay at the NICU (Blauw-Hospers and Hadders-Algra, 2005; Saccani et al., 2013; Mallmann et al., 2023) may be harmful to early development. At the NICU, the newborn is manipulated approximately 40 to 130 times in a 24-h period, being exposed to several adverse procedures (Gottfried and Hodgman, 1984; Aita and Goulet, 2003; Nicolau et al., 2011). Intense and continuous luminosity, noise, and separation from the mother are added. Furthermore, as very preterm newborns are usually born with very low weight, they are exposed to illness and can struggle to develop in the NICU. Hence, although hospitalization is essential for the survival of very preterm newborns, it can be accompanied by a set of excessive stimuli in a challenging environment and physiological context (Munhoz et al., 2022; Rees et al., 2022). Considering our results and the literature, it is likely that the adversities surrounding neonatal hospitalization and low birth weight can be additional barriers to learning to reach and grasp in very preterm infants. As this is the first study that addresses the relationship between such clinical variables and reaching, and as we did not measure it directly, this topic deserves further investigation in future research to better target promotion and prevention measures in this field.

It should be emphasized that age correction for preterm birth could have nullified disadvantages between infants of different gestational age groups in relation to the period of reaching emergence (Soares et al., 2014). This was not observed in the present study as late preterm infants reached earlier than very preterm infants independent of age correction. However, it is important to stress that as the assessments were carried out in the same stage of skill maturity in both groups (a few days after the emergence of reaching), our results express the performance of infants with the same level of reaching skill, regardless of age. In fact, there were no differences between the infants with respect to the number of total reaches, which reinforces the similarity in the skill level between the groups. This corroborates that differences in grasping between the groups were not caused by differences in skill level or age adjustment. This is also supported by the permanence of the results between the groups even when controlling for age at reaching onset as an intervening variable.

Based on our findings and considering that reaching skills may predict neurodevelopment at 2 years in infants born very preterm (Kaul et al., 2019), we recommend assessment and stimulation of reaching and grasping behaviors to be regarded as part of follow-up and early intervention programs. This could be adopted as a strategy to monitor early manual skills and prevent reaching, grasping, and, perhaps, other developmental problems, especially in very preterm infants.

We recognize several limitations in this study. The small sample size is a major issue and may have increased the margin of error in the results. In addition, it may not be representative of the population of preterm infants, compromising the external validity of the study. It is also not possible to ensure the quality of the measurement of the clinical variables considered as they were obtained from secondary sources. Furthermore, we do not know whether the differences shown between the infants are just transitory as they were not assessed in subsequent months. Therefore, caution is important in interpreting our findings. As far as we know, this is the first study to analyze differences in motor behavior according to the degree of prematurity. It is noteworthy that the results suffered little interference from the infants’ skill level as the acquisition of reaching was monitored so that the assessments occurred soon after the emergence of the first reaching movements.

In general, this study shows that this topic deserves extended investigation in future research. Carrying out longitudinal cohort studies with larger sample sizes will be important to address our findings and interpretations further. Future studies could also investigate whether other common neonatal clinical features, such as the use of mechanical ventilation and peri-intraventricular hemorrhage, can affect early reaching behavior in preterm infants. A third comparative group with full-term infants could shed more light on the role of the degree of prematurity in reaching. In addition, future research could investigate the influence of the toys’ characteristics on reaching and grasping among the preterm infant population.

## 5. Conclusion

A higher degree of prematurity was unfavorable to the acquisition of manual reaching and the performance of reaches with grasping, considering very preterm infants compared with late preterm infants. This was influenced by the lower birth weight and longer length of hospital stay in the very preterm group.

## Data availability statement

The raw data supporting the conclusions of this article will be made available by the authors, without undue reservation.

## Ethics statement

The study was approved by the Research Ethics Committee of the Federal University of Mato Grosso do Sul. The study was conducted in accordance with the local legislation and institutional requirements. Written informed consent for participation in this study was provided by the participants’ legal guardians/next of kin. Written informed consent was obtained from the minor(s)’ legal guardian/next of kin for the publication of any potentially identifiable images or data included in this article.

## Author contributions

AF: Methodology, Writing – review & editing, Data curation, Writing – original draft. PT: Data curation, Writing – original draft, Writing – review & editing. AO: Formal Analysis, Writing – review & editing. ET: Writing – review & editing. DS-M: Conceptualization, Formal Analysis, Funding acquisition, Methodology, Project administration, Supervision, Writing – review & editing.
